# Colistin Resistance in *Acinetobacter baumannii* Clinical Isolates from Bahrain: Evaluation of Detection Methods and Clonal Relationships

**DOI:** 10.3390/antibiotics15060532

**Published:** 2026-05-23

**Authors:** Zainab Husain Salman, Mohd Shadab, Zainab Salman Saleh, Nouf Al-Rashed, Mohammad Shahid

**Affiliations:** 1 Department of Microbiology, Immunology and Infectious Diseases, College of Medicine and Health Sciences, Arabian Gulf University, Manama P.O. Box 26671, Bahrain; 2 Al-Sabah Hospital Laboratories, Al-Sabah Health Region, Kuwait City P.O. Box 4078, Kuwait; noufmallrashed@moh.gov.kw

**Keywords:** *Acinetobacter*, *A. baumannii*, colistin, fingerprinting, AST, *mcr*, broth microdilution method, colistin agar test, CRAB, antimicrobial resistance

## Abstract

**Background**: *Acinetobacter baumannii* (*A. baumannii*) is a critical-priority pathogen of major concern in healthcare settings. Colistin remains a last-resort antibiotic for multidrug-resistant (*MDR*) *A. baumannii* infections; however, resistance is increasingly reported worldwide yet remains understudied in Bahrain. Reliable detection methods and understanding clonal dissemination are essential for infection control. **Objectives:** This study aimed to (1) determine the rate of colistin resistance in 102 clinical *A. baumannii* isolates from Bahrain, (2) evaluate the diagnostic performance of the colistin agar test (CAT) and E-test against broth microdilution (BMD method), and (3) assess clonal relationships using BOX-PCR fingerprinting. **Methods**: 102 clinical isolates from multiple hospitals in Bahrain underwent susceptibility testing via the BMD method, CAT, and E-test; screening for *mcr-1* to *mcr-5* genes; and BOX-PCR DNA fingerprinting. **Results**: Colistin resistance was detected in 14.7% of isolates by BMD method, higher than regional and global averages. All resistant isolates were *mcr*-negative, suggesting chromosomally mediated resistance. CAT showed 86.7% sensitivity, 98.8% specificity, and a 13.3% very major error rate. The E-test failed to detect resistant isolates (very major error 100%). BOX-PCR revealed predominant clonal relatedness with intra- and inter-hospital spread. **Conclusions**: Colistin resistance in *A. baumannii* from Bahrain exceeds regional and global levels, likely driven by chromosomal mechanisms under selective pressure. The BMD method remains the gold standard for colistin testing, while CAT may serve as a screening tool requiring confirmation. Strengthened stewardship and infection control measures are vital to contain dissemination.

## 1. Introduction

*Acinetobacter baumannii* (*A. baumannii*) is a Gram-negative, aerobic, glucose-non-fermentative, non-motile coccobacillus [1]. This organism can form biofilms on surfaces like medical devices, allowing it to survive and persist in hospital environments and increase the risk of causing healthcare-associated infections. Additionally, it can acquire resistance to multiple antibiotics leading to more challenges in treatment [2]. Antibiotic resistance in this organism has been reported worldwide, with multidrug-resistant (MDR) *A. baumannii* strains being resistant to at least one agent in three or more classes of the antimicrobial agents, including beta-lactams (such as carbapenems and cephalosporins), aminoglycosides, and fluoroquinolones [3].

The increasing incidence of MDR *A. baumannii* is challenging and threatening. This organism can lead to high morbidity and mortality rates in patients, especially those in intensive care units (ICUs) [4]. Infections with MDR *A. baumannii* can present as hospital-acquired pneumonia, bloodstream infections, and wound, urinary tract, meningitis, endocarditis, and soft tissue infections [3]. The inability to provide appropriate antibiotic treatment due to multidrug resistance may lead to longer lengths of stay in a hospital and higher mortality rates. Mortality rates from infection with MDR *A. baumannii* is above 35% in ICUs and can reach up to 50% in bloodstream infections [5]. The World Health Organization (WHO) has classified carbapenem-resistant *A. baumannii* as a critical-priority pathogen requiring new antibiotics research and development because of its global threat [6].

Colistin (polymyxin E) is from the polymyxin class of antibiotics. It is used to treat infections with MDR strains [7]. Colistin targets Gram-negative bacteria and is considered bactericidal by damaging the outer membrane through interacting with the bacterial lipopolysaccharide (LPS). Colistin is a positively charged molecule that interacts with the negatively charged groups of lipid A of LPS by displacing the divalent Ca^2+^ and Mg^2+^ and eventually creates disruption of the membrane. By interacting with LPS, colistin also neutralizes the LPS endotoxin activity. Colistin also causes the intracellular accumulation of reactive oxygen species and induces oxidative damage [8].

Despite colistin having highly effective antimicrobial activity, there are still concerns regarding its nephrotoxicity, which restricts its use as the initial drug of choice when the isolate is susceptible to other antibiotics. Consequently, colistin is considered a “last resort” antibiotic only for MDR strains and extensively drug-resistant strains (XDR strains) [9].

Resistance to colistin arises due to several mechanisms that result in the loss or modification of the LPS, reducing the ability of colistin to bind to the cell. This resistance can be chromosomally encoded, such as (1) the loss of lipopolysaccharide (LPS) structure due to mutations in LPS biosynthetic genes (*lpxA*, *lpxC*, *lpxD*); (2) the addition of phosphoethanolamine (PEtN) moiety, which reduces the overall negative charge of lipid A, weakening the electrostatic attraction between colistin and the bacterial outer membrane, mediated by the *pmrCAB* operon or *eptA* gene; and (3) the upregulation of efflux pump genes (e.g., *EmrAB* and *AdeIJK*), which encode for proteins that expel colistin out of the cell. On the other hand, there are plasmid-encoded mechanisms such as the mobile colistin resistance genes (*mcr* genes), which were first discovered in *Escherichia coli* (*E. coli*) in China in 2015. Among the *mcr* genes, *mcr-1* and *mcr-4.3* are the most detected variants in *A. baumannii*. They encode PEtN transferases that enzymatically modify lipid A in LPS [10,11].

The phenotypic detection of colistin resistance is crucial for the appropriate treatment of patients. For this, the broth microdilution test (BMD method) is considered the gold standard for colistin susceptibility testing. However, it is labor-intensive and not available in every laboratory. The colistin agar test (CAT) and gradient diffusion (E-test) are other methods for colistin susceptibility testing, but these methods are not recommended by the Clinical and Laboratory Standards Institute (CLSI) or the European Committee on Antimicrobial Susceptibility Testing (EUCAST) due to their inaccuracy [12]. The CLSI and EUCAST have classified the BMD method as the only recommended method for colistin MIC testing [13].

There are differences in the prevalence of colistin resistance in *A. baumannii* around the world. Colistin resistance rates have increased from 2% pre-2011 to 4% post-2012 [14]. The most significant reports of *A. baumannii* colistin resistance arise from regions that have high polymyxin usage rates like France and the United Arab Emirates [14]. Middle Eastern countries typically report higher rates of colistin resistance—for example, 17.5% in Lebanon, and over 70% in some hospitals in Iraq—which shows the burden of colistin resistance in this region [15,16]. Currently, there is a gap in the knowledge of colistin resistance among *A. baumannii* isolates in the Kingdom of Bahrain.

Therefore, this study aims to (1) determine the rate of colistin resistance in clinical *A. baumannii* isolates from Bahrain, (2) evaluate the diagnostic accuracy of the CAT and E-test by comparing their results with the broth microdilution test, and (3) assess the clonal relationship among resistant isolates using BOX DNA fingerprinting.

## 2. Results

### 2.1. Isolate Distribution

**Figure 1:**
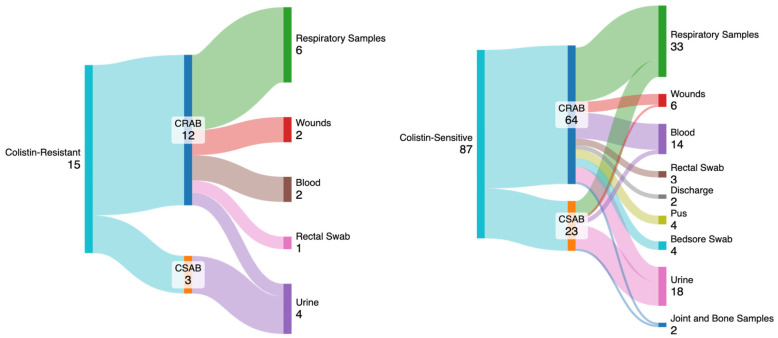
Sankey diagrams showing distribution of all isolates by colistin resistance (**left**) and colistin susceptibility (**right**) with their clinical sample type. CRAB = carbapenem-resistant *A. baumannii*, CSAB = carbapenem-sensitive *A. baumannii*.

Of the 102 *A. baumannii* isolates, 15 (14.7%) isolates were colistin-resistant based on the broth microdilution test. The distribution of colistin resistance across the different types of samples is shown in Figure 1.

Of these, 76 (74.5%) were carbapenem-resistant (CRAB) and 26 (25.5.3%) were carbapenem-sensitive (CSAB). Colistin resistance was observed more in respiratory samples (*n* = 6), followed by urine (*n* = 4), while the lowest rates of resistance were observed in blood samples (*n* = 2), wound infections (*n* = 2), and rectal swabs (*n* = 1).

### 2.2. Evaluation of Colistin Susceptibility Methods

Major error (ME), very major error (VME), categorical agreement, sensitivity, and specificity were the characteristics used to evaluate the diagnostic effectiveness of the colistin agar test (CAT), E-test and the BMD method [17] Among the 102 isolates, 15 (14.7%) were identified as *Col-R* by the BMD method. CAT identified 13 out of 15 resistant isolates (86.7%) when tested on the 102 isolates. However, CAT still demonstrated a VME rate of 13.3%, which is above the CLSI’s accepted threshold (≤1.5%). The E-test did not detect any *Col-R* isolates and had a VME rate of (100%), which indicates poor sensitivity in detecting colistin resistance a very high very major error (VME) rate (100%).

A summary of the detailed comparative performance metrics, which consist of categorical agreement, sensitivity, specificity, and error rate, can be found in Table 1.

The E-test was only performed on the 15 isolates identified as resistant by the BMD method. Therefore, specificity, major error (ME), and categorical agreement could not be determined for the E-test.

BMD was used as the reference method. Categorical agreement (CA) was calculated as the number of isolates with the same categorical result by the test method and reference method divided by the total number of isolates tested × 100. Very major error (VME) was calculated as the number of resistant isolates by the reference method that were falsely classified as non-resistant by the test method divided by the total number of resistant isolates by the reference method × 100. Major error (ME) was calculated as the number of non-resistant isolates by the reference method that were falsely classified as resistant by the test method divided by the total number of non-resistant isolates by the reference method × 100. For CAT, 13/15 BMD-resistant isolates were classified as resistant and 2/15 were classified as non-resistant, giving a VME of 13.3%. Among the 87 BMD-non-resistant isolates, 0/87 were falsely classified as resistant by CAT, giving an ME of 0%. Categorical agreement was calculated as (13 + 87)/102 × 100 = 98.0%. CLSI 2024 interpretive criteria were applied, with colistin MIC ≥ 4 µg/mL interpreted as resistant [18].

### 2.3. PCR Screening for mcr Genes

All 15 *Col-R A. baumannii* isolates were negative for all *mcr* genes (i.e., *mcr-1* to *mcr-5*). This suggests that colistin resistance is not due to any plasmid-mediated mechanism.

### 2.4. Determination of Clonal Relationship by Dice-UPGMA Analysis

**Figure 2:**
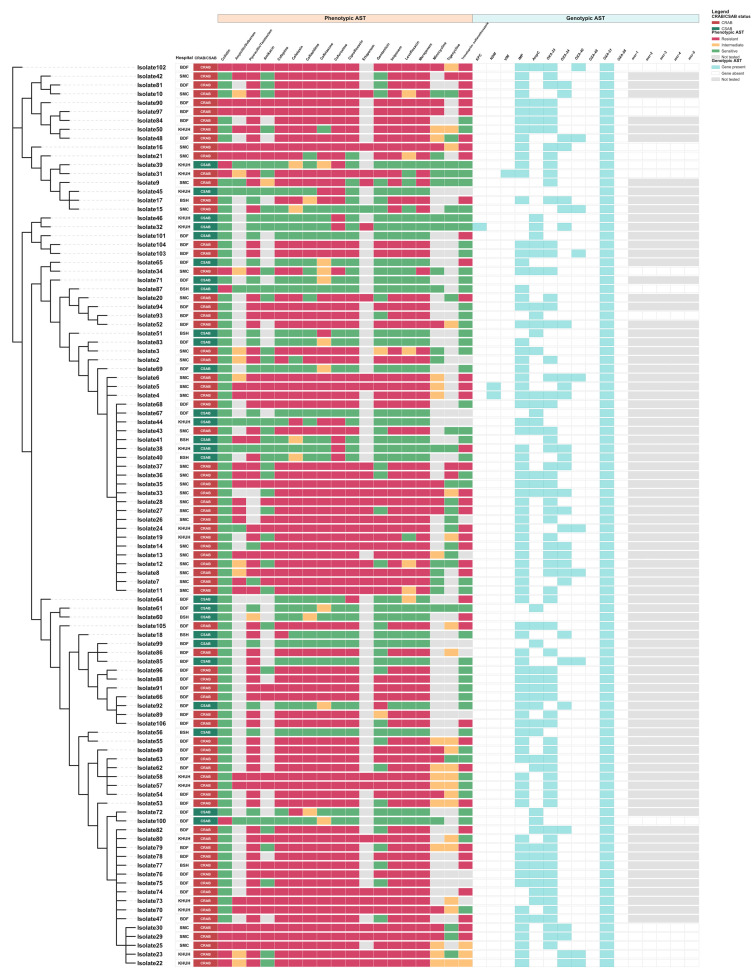
BOX-PCR fingerprinting with phenotypic and genotypic AST profile of 102 *A. baumannii* isolates. (**Left**): Dendrogram created from the BOX-PCR patterns using the UPGMA method and Dice similarity coefficient. (**Right**): Heatmap showing hospital origin, CRAB/CSAB status, antimicrobial susceptibility profiles across 18 antibiotics, and genotypic AST.

The relationship among the *A. baumannii* isolates was determined through dice similarity coefficients and represented with UPGMA clustering, as shown in Figure 2. After clustering, the isolates were compared in regard to their source (hospital name), CRAB vs. CSAB status, and phenotypic and genotypic AST profiles. This method yielded three major clusters. The third cluster contained >65% of isolates from one hospital with several subclusters showing an identical profile (0% genetic distance). Furthermore, across all clusters, numerous isolates had very small genetic distances and some were identical. Generally, isolates within each cluster shared similar phenotypic and genotypic AST.

*Col-R* isolates were present in all three clusters: seven in cluster 1, two in cluster 2, and six in cluster 3. The *Col-R* isolates were from all four hospitals. In cluster 1, all *Col-R* isolates, except one, had genetic distance of <0.1, with two being identical. The *Col-R* isolates in cluster 2 had a genetic distance of >0.1. In cluster 3, all *Col-R* isolates had genetic distance of <0.1 and five of the isolates were identical. All *Col-R* isolates had similar genotypic and phenotypic AST profiles. No large hospital-specific cluster was observed among the *Col-R* isolates. However, in both clusters 1 and 3, some identical *Col-R* isolates were found to be from the same hospital. In all of these Col-R isolates, the most effective antibiotics were amikacin (seven susceptible), minocycline (six susceptible, three intermediate), and tigecycline (five susceptible, two intermediate, and no resistant isolates). Other antibiotics included ceftazidime (five susceptible) and fluoroquinolones (ciprofloxacin and levofloxacin, of which five isolates were susceptible), trimethoprim–sulfamethoxazole (four susceptible), tobramycin and gentamycin (four susceptible), ampicillin–sulbactam (two susceptible, four intermediate), and ampicillin–tazobactam (two susceptible isolates). Regarding their genotypic AST, *bla*_KPC_ and *bla*_NDM_ were not detected. *bla*_VIM_ was identified in one isolate (6.7%), *bla*_IMP_ in all isolates (100%), *bla*_OXA-23_ in 11 isolates (73.3%), *bla*_OXA-24_ in five isolates (33.3%), and *bla*_OXA-40_ in three isolates (20%), while *bla*_OXA-48_ was not found in any of the isolates (0%) and *bla*_OXA-58_ was found in all isolates (100%).

## 3. Discussion

In our isolates, 15 out of 102 (14.7%) clinical *A. baumannii* isolates were colistin-resistant according to the BMD method, which is above the global average of 4–5% [14]. This also exceeds the rates reported in the neighboring Gulf Cooperation Council (GCC) countries, including the Kingdom of Saudi Arabia (9.3%), United Arab Emirates (less than 4%) [19], Kuwait (12%) [20], and Qatar (1.4%) [21]. Multiple factors may explain the high level of colistin resistance seen in our *A. baumannii* isolates. Although the study was not designed to estimate national prevalence, the resistance rate observed among these isolates appears higher than that reported in some previous regional and international studies; therefore, it should be interpreted as a notable multicenter observation rather than a definitive national epidemiological estimate. This might be due to the inappropriate use of colistin in both human and veterinary environments that may have resulted in selective pressure that favored the *Col-R* strains to survive. Colistin is used as a “last-resort” antibiotic in hospitals, but it had been previously used across many countries, including GCC countries, as a growth-promoting and prophylactic agent in livestock [22] Bahrain officially banned colistin use in animal farming in 2024 [23]. This is considered a long period of time that may allow resistant strains to enter clinical settings by environmental spread or the transmission of resistant strains from animals. The unregulated use of colistin also might be a reason for the spread of these strains in the environment and food chain. In addition to selective pressure, recent evidence indicates that colistin resistance may also be an adaptive response to oxidative stress with mutations that allow for survival under membrane-damaging conditions associated with colistin exposure [24,25].

The clonal dissemination of resistant strains in healthcare settings is likely contributing to the spread of the *Col-R* strains. *A. baumannii* is known for causing hospital outbreaks by highly transmissible and resistant clones, such as ST2 (International Clone II). This is a globally disseminated clone that is often associated with colistin and carbapenem resistance [26]. The clustering seen in our dendrogram using BOX-PCR also indicates nosocomial transmission.

For the performance of the colistin agar test (CAT) and E-test compared to the BMD method, CAT detected 13/15 resistant isolates with a sensitivity of 86.7% and specificity of 100% and a very major error (VME) rate of 13.3% (the CLSI threshold is <3% VME). The previous studies that evaluated the accuracy of CAT reported a sensitivity and a specificity of between 94 and 100% with a VME less than 6% [27]. Additionally, CAT had a much lower VME (13.3% vs. 100% for E-test), making it a safer option than the E-test to screen for colistin resistant isolates. Additionally, CAT is less expensive and easier to perform and interpret in comparison to the BMD method, which is useful for laboratories that have limited resources available to perform the BMD method.

For the two resistant isolates, CAT was not able to detect the Col-R isolates. This could be attributed to heteroresistance, where a small percentage of a susceptible isolate is resistant to colistin. When they come under colistin pressure, these subpopulations emerge from the colistin-susceptible population [28]. The small population may not grow on CAT and can give a false negative result. Heteroresistant populations often appear as “skipping wells” in the BMD method dilution series, which was also observed in our BMD method in both isolates that were missed by CAT. It is described as growth in a skip-well in broth, which might indicate heteroresistance; however, agar methods (like CAT) that use only a concentration of colistin can miss this altogether and lead to false negative results [28].

In contrast, the colistin E-test was not able to identify any of the resistant colistin strains, resulting in a sensitivity of 0% (VME rate 100%). This is consistent with other data that have shown the unreliability of the E-test. For example, a previously published study found a VME rate of 35% for the E-test in the detection of colistin resistance [29]. Similarly, a study in India demonstrated that the E-test had a categorical agreement of 78% with a VME of 21.9%, which is an unacceptable VME above the thresholds [30]. EUCAST has warned against using these methods for polymyxins [31] as the misclassification of resistant isolates as susceptible may lead to ineffective therapy and nosocomial transmission.

In this study, all colistin-resistant *A. baumannii* isolates were negative for *mcr* genes, which is consistent with other published evidence showing that these genes are rare in *A. baumannii* and more common in Enterobacteriaceae [32]. The absence of *mcr* genes in our population suggest that colistin resistance mechanisms for our isolates might be chromosomally mediated, which is the most common mechanism responsible for colistin resistance as per NCBI MicroBIGG-E Map [33].

Though the management of *Col-R A. baumannii* is difficult, especially if it is also carbapenem-resistant, The Infectious Diseases Society of America (IDSA) guidelines recommend sulbactam–durlobactam in combination with a carbapenem (meropenem or imipenem–cilastatin) as the preferred treatment regimen for CRAB infections [34]. This approach was recently approved by the FDA for the treatment of hospital- and ventilator-associated pneumonia related to CRAB. The Phase 3 ATTACK (Acinetobacter Treatment Trial Against Colistin) trial showed that Sul-Dur in combination with imipenem was better than colistin–imipenem for patients with severe CRAB infections. Although it has recently been shown to be a promising alternative for *Col-R* strains, it was able to achieve activity against OXA-type carbapenemases with a lower mortality (19.0% vs. 32.3%), higher clinical cure rate (61.9% vs. 40.3%) and significantly less nephrotoxicity (reported as 13.2% vs. 37.6%) compared to colistin [35]. In the absence of sulbactam–durlobactam, high-dose ampicillin–sulbactam in combination with at least one other active agent is also recommended [34]. Minocycline or tigecycline are second-line alternatives, only used when the isolate is susceptible, usually with sulbactam or polymyxins (if still susceptible). Cefiderocol (a new cephalosporin with activity against OXA carbapenemase producers) could be another option for CRAB. There are guidelines that specifically state that cefiderocol should be used in combination as a treatment and not recommended as a monotherapy for *A. baumannii* regimens [36].

Based on our study, most *Col-R* isolates were also CRAB with MDR/XDR AST profiles. As per these findings, the empiric backbone may include sulbactam–durlobactam in combination with a carbapenem as per the guidelines [34], or, if novel agents are unavailable, our findings suggest that high-dose ampicillin–sulbactam may not be reliable as monotherapy (only two isolates were susceptible in our *Col-R* isolates) but may still be useful in combination because of sulbactam’s intrinsic activity against *A. baumannii* and synergy with the other agent [34]. In our case, empiric combinations such as ampicillin–sulbactam plus a tetracycline (e.g., minocycline or tigecycline) or ampicillin–sulbactam plus or an aminoglycoside (amikacin or gentamicin if not contraindicated) may be considered depending on the site of infection. Based on our findings, relying on colistin monotherapy as empiric therapy may be unsafe even though the majority of isolates are considered susceptible. However, evidence also shows that certain colistin-based combinations may still offer some help before AST results are available. The most reliable results have been seen with sulbactam-containing regimens (now augmented by sulbactam–durlobactam) [37]. Combining colistin with high-dose sulbactam (9 g sulbactam/day) has been associated with improved early survival [38]. Colistin combinations with tigecycline or minocycline can also improve clinical outcomes if given in high doses. On the other hand, adding a carbapenem to colistin has not improved patient outcomes in trials [37].

## 4. Materials and Methods

### 4.1. Sample Collection and Bacterial Identification

A total of 102 *A. baumannii* isolates were procured from a previous study performed by another research group but with different objectives [16]. These samples were collected from the major hospitals in Bahrain. Identification of the bacteria and antibiotic susceptibility testing (AST) was conducted by automated identification systems, e.g., VITEK 2 (bioMerieux, Marcy-l’Étoile, France) and BD Phoenix [16].

Colistin susceptibility testing was carried out using three different methods, i.e., the gradient strip test (E-Test), colistin agar test (CAT), and broth microdilution (BMD) method, to compare and evaluate the findings as CLSI only recommends broth microdilution for colistin susceptibility testing.

### 4.2. Broth Microdilution Method

A broth microdilution test was performed to determine the minimum inhibitory concentration (MIC) of colistin for each isolate.

The inoculum was prepared by suspending freshly grown colonies in 0.45% saline. The suspension was adjusted to match a turbidity level to 0.5 McFarland standard using a Densicheck apparatus (BioMerieux, Marcy-l’Étoile, France). This suspension was then diluted 1:150 in Mueller Hinton Broth (MHB) to achieve colony counts of approximately 1 × 10^6^ CFU/mL.

Then, 96-well microdilution plates were set up, with each well containing 50 µL of MHB and a serial twofold dilution of colistin, ranging from 64 to 0.5 µg/mL. Then, 50 µL of bacterial suspension was added into each well to reach a final concentration of approximately 5 × 10^5^ CFU/mL following the guidelines outlined by CLSI. The plates were incubated at 37 °C for 18–24 h. MICs were determined as the lowest concentration of colistin where no visible growth was detected. As per CLSI recommendations, isolates with an MIC of ≤2 mg/L were considered intermediate, and those with an MIC of ≥4 mg/L were considered resistant [18]. The negative control used was *A. baumannii* ATCC 19606, while *Providencia stuartii* (intrinsically resistant) was used as a positive control.

### 4.3. E-Test

For the E-test, the 0.5 McFarland suspension was streaked with a sterile cotton swab in 4 directions onto MHA plates, and colistin AST strips (Bioanalyse, Ankara, Turkey) with concentrations ranging from 0.016 to 256 µg/mL were placed on the surface. The plates were incubated at 37 °C for 18–24 h. MICs were determined as the concentration where the inhibition of growth intersected with the strip.

### 4.4. Colistin Agar Test

A colistin drug suspension was prepared by dissolving 10 mg of colistin sulphate powder (Sigma-Aldrich, St. Louis, MO, USA) in 10 mL of sterile water to obtain a concentration of 1 mg/mL. Colistin agar plates were prepared by adding 0.4 mL of the 1 mg/mL colistin antibiotic suspension into 100 mL of MHA, and maintained at 45 °C, to achieve a final concentration of 4 μg/mL. This mixture was poured into Petri dishes and solidified. A sterile cotton swab was dipped into the 0.5 McFarland bacterial suspension and a spot was applied on the colistin agar surface, and the plates were incubated at 37 °C for 18–24 h. The isolate was considered colistin-resistant (*Col-R*) if visible growth was seen.

### 4.5. Detection of mcr Genes via Polymerase Chain Reaction (PCR) Screening

**DNA extraction:** The DNA of the bacterial isolates was extracted from a pure culture using the heat-and-chill method. Bacterial colonies were suspended in 500 μL of TE buffer, boiled for 10 min, and then cooled on ice for 5 min. The sample was centrifuged at 10,000 rpm for 10 min. The supernatant was stored in fresh tubes.

**Primer designing:** After thorough investigation on PubMed and as per the MicroBIGG-E Map, only mcr-1 and mcr-4 have been detected in *A. baumanii* around the world [33]. Hence, NCBI Primer BLAST tool was used to design the primers for the two genes [39]. Two universal primer sets were generated for the *mcr-1* gene (labeled as *mcr-1.1U* and *mcr-1.2U*). Primers were generated at the extra flanking sequences of the gene covering the full gene length of 1626 bp. The same approach was applied for designing the universal primer set of *mcr-4* gene (1626 bp). This primer set was also designed at the extra flanking consensus region to amplify all of the alleles of the mcr-4 gene. Another screening primer set was created to amplify a short but highly conserved sequence of all *mcr-4* subtypes (9 alleles) capable of amplifying any subtype of *mcr-4* [40].

**Polymerase chain reaction (PCR**): Conventional PCR was conducted on the *Col-R* isolates to screen for the presence of *mcr-1* and *mcr-4* genes using self-designed primers after standardizing through the gradient PCR method. For this, 3 µL of the DNA was used as a template along with 12.5 µL of Master Mix (Promega, Madison, WI, USA), 7.5 µL of nuclease-free water, and 1 µL of each primer. Additionally, a multiplex PCR approach was also adopted to screen these isolates for the presence of *mcr-1*, *mcr-2*, *mcr-3*, *mcr-4*, and *mcr-5* genes from Rebelo et al. (2018) [41]. This multiplex PCR included 2.5 μL of Master Mix, 5.5 μL of nuclease-free water, 0.5 μL of each of the 10 primer solutions (10 μM conc.), and 2 μL DNA template. Details of the primer sequences and cycle conditions are shown in Table 2.

### 4.6. DNA Fingerprinting by BOX-PCR

After phenotypic testing, the 102 isolates were subjected to BOX DNA fingerprinting [42,43]. PCR was performed with a final volume of 25 µL. PCR reaction included 12.5 µL of Master Mix (Promega, Madison, WI, USA), 4 µL of primer (Eurofins, Nantes, France), 2 µL of DNA template, and 6.5 µL of nuclease-free water. The primer sequences and PCR cycle conditions that were used to amplify the BOX genes are shown in Table 2.

After PCR, gel electrophoresis was carried out in 1% agarose gel. The bands were visualized using a Gel-doc system (C200 Azure Biosystems, Dublin, CA, USA).

To analyze the results, each isolate lane was transformed into a binary matrix. The genetic similarity among samples was calculated using the Dice coefficient method. The unweighted pair group method with arithmetic mean (UPGMA) was performed to cluster the strains and to create a phylogenetic tree using insilico webtool [44].

The strains that clustered at a genetic distance of <0.1 (>90% similarity) were considered to be clonally related, with reference to previously reported cutoffs for the genotypic fingerprinting of *A. baumannii* using Dice-based UPGMA dendrograms for DNA fingerprinting [40]. The genotypic and phenotypic AST data were retrieved from the study by Nouf et al. [16].

## 5. Conclusions

Among clinical *A. baumannii* isolates from four major hospitals in Bahrain, colistin resistance was detected in 14.7% of isolates, representing a clinically relevant multicenter local finding rather than a formal national prevalence estimate. This rate is higher than the regional and global average. BOX-PCR revealed clonal relationships and suggested the spread of MDR *A. baumannii* within individual hospitals and between different hospitals. Within this population, the colistin-resistant isolates were also predominantly clonally related. There was no dominant hospital-specific colistin-resistant cluster, but there were some identical isolates within the same hospital, which suggests intra-hospital transmission as well as inter-hospital spread. All of the colistin-resistant isolates tested negative for *mcr-1*, *mcr-2*, *mcr-3*, *mcr-4*, and *mcr-5*, which suggests chromosomally mediated resistance. For diagnostics, BMD should remain the gold standard. The E-test is not a reliable method for polymyxin susceptibility testing, and CAT may be a cost-effective screening test for low-resource laboratories but may require confirmatory testing using BMD. Controlling the spread will require coordinated efforts across hospitals to limit colistin use and to strengthen infection control.

## Figures and Tables

**Table 1:** Performance metrics of colistin susceptibility testing methods (*n* = 102 isolates).

Method	Resistant Isolates Detected	Categorical Agreement (%)	Sensitivity (%)	Specificity (%)	VME %	ME %
**BMD method**	15	NA	NA	NA	NA	NA
**CAT**	13	98	86.7	100	13.3	0
**E-test**	0	NA	0	NA	100	NA

Abbreviations: VME, very major error (resistant isolates falsely classified as susceptible; CLSI threshold ≤ 1.5%); ME, major error (susceptible isolates falsely classified as resistant; CLSI threshold ≤ 3%) [17].

**Table 2:** Primer sequences and conditions used to amplify *mcr* genes and BOX fingerprinting.

Primer	Primer Sequence 5′ to 3′	PCR Conditions	Amplicon Size (bp)	Reference
**BOXA1R**	CTACGGCAAGGCGACGCTGACG	94° C for 5 min, followed by 35 cycles at 94° C for 1 min, 36° C for 1 min, 72° for 3 min, and a final extension at 72° for 10 min:	NA	[42]
***mcr-1.1U***	Forward: ATTGCCGCAATTATCCCACC Reverse: CACCGCCCATAATACGAATG	94° C for 5 min, followed by 30 cycles at 94° C for 1 min, 53.5° C for 1 min, 72° C for 2 min, and a final extension at 72° C for 10 min	1726	Self-designed
***mcr1.2U***	Forward: CAGATAAATTGTACTGGATTTC Reverse: CACCGCCCATAATACGAATG	94° C for 5 min, followed by 30 cycles at 94° C for 1 min, 52° C for 1 min, 72° C for 2 min, and a final extension at 72° C for 10 min	1766	Self-designed
***mcr-4U***	Forward: TAATGAGGTCAAGCTAGT Reverse: GACATTGTTAGTCCAAGAT	94° C for 5 min, followed by 30 cycles at 94° C for 1 min, 49° C for 1 min, 72° C for 2 min, and a final extension at 72° C for 10 min	1741	Self-designed
***mcr-4S***	Forward: GTTGTGGGTGAAACTGC Reverse: TCACACACACCTTTACAGC	95° C for 5 min, followed by 30 cycles at 95° C for 1 min, 50.4° C for 1 min, 72° C for 50 s, and a final extension at 72° C for 5 min	281	Self-designed
**Primers for multiplex PCR for *mcr* genes detection**				
***mcr-1***	Forward: AGTCCGTTTTTTTGTGGC Reverse: AGATCCTTGGTCTCGGCTTG	94 °C for 15 min, followed by 25 cycles at 94 °C for 30 s, 58 °C for 90 s and 72 °C for 60 s, and a final extension at 72 °C for 10 min.	320	[41]
***mcr-2***	Forward: CAAGTTGTTGGTCGCAGTT Reverse: TCTAGCCCGACAAGCATACC		715	
***mcr-3***	Forward: AAATAAAAATTGTTCCGCTTATG Reverse: AATGGAGATCCCCGTTTTT		929	
***mcr-4***	Forward: TACTTTCATCACTGCGTTG Reverse: TTGGTCCATGACTACCAATG		1116	
***mcr-5***	Forward: ATGCGGTTGTCTGCATTTATC Reverse: TCATTGTGGTTGTCCTTTTCTG		1644	

## Data Availability

The original contributions in this study are included in the article. Further inquiries can be directed to the corresponding author.
